# Extracellular vesicles derived from lung M2 macrophages enhance group 2 innate lymphoid cells function in allergic airway inflammation

**DOI:** 10.1038/s12276-025-01465-6

**Published:** 2025-06-02

**Authors:** Kun Lv, Yingying Zhang, Guoquan Yin, Xueqin Li, Min Zhong, Xiaolong Zhu, Weiya Pei

**Affiliations:** 1 Central Laboratory, The first affiliated hospital of Wannan Medical College, Wuhu, People’s Republic of China; 2 Anhui Province Key Laboratory of Non-coding RNA Basic and Clinical Transformation (Wannan Medical College), Wuhu, People’s Republic of China; 3 Clinical Research Center for Critical Respiratory Medicine of Anhui Province, Wuhu, People’s Republic of China; 4 Department of Laboratory Medicine, The first affiliated hospital of Wannan Medical College, Wuhu, People’s Republic of China; 5 Yangzhou Blood Center, Yangzhou, People’s Republic of China

**Keywords:** Innate lymphoid cells, Extracellular signalling molecules

## Abstract

Group 2 innate lymphoid cells (ILC2s) promote the recruitment of eosinophils by secreting large amounts of type 2 cytokines (IL-5 and IL-13), thus triggering the main feature of asthma, pathological inflammation. Recent insights from mouse and human studies indicated a potential relationship between ILC2s and macrophages. However, the mechanism by which lung M2 macrophage-derived extracellular vesicles (M2 EVs) regulate ILC2s remains unclear. Here the size, morphology and specific markers of M2 EVs were successfully characterized in the lungs. Furthermore, we discovered that M2 EVs strongly promoted type 2 immune inflammation induced by papain. Mechanistically, M2 EVs were internalized by ILC2s, triggering ILC2s activation and inducing pro-inflammatory cytokine (IL-5 and IL-13) production. M2 EVs also indirectly enhanced the function of ILC2s through macrophages and CD4^+^ T cells. Using RNA sequencing, we found that long non-coding RNA 4930474H06Rik participated in mediating these effects of M2 EVs. Further mechanistic studies have elucidated the underlying mechanism by which 4930474H06Rik influences the function of ILC2s. Inhibition of 4930474H06Rik significantly altered the intracellular metabolic status of activated ILC2s and effectively alleviated airway inflammation in a mouse model of asthma. Taken together, we demonstrated that M2 EVs promoted allergic airway inflammation at least partially through 4930474H06Rik, implying that 4930474H06Rik can be considered as a therapeutic target for ILC2s activation in allergic airway inflammation.

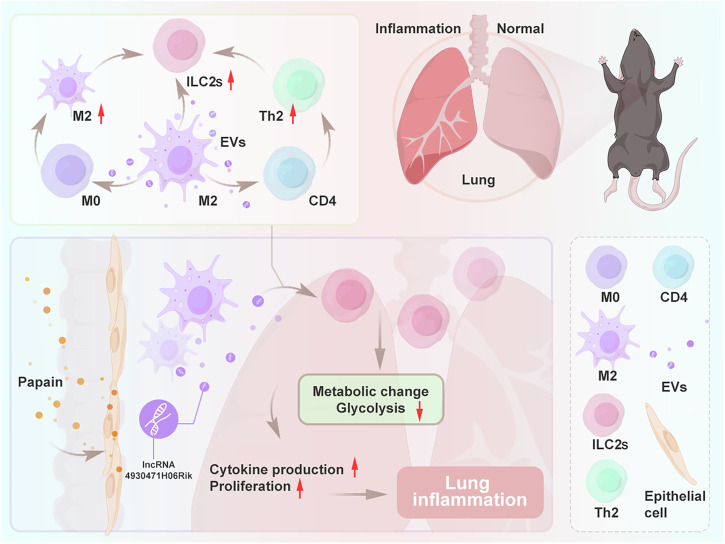

## Long non-coding RNA enhances ILC2s activation in asthma

Asthma is a common lung disease that causes breathing problems. Researchers are trying to understand how certain immune cells, such as M2 macrophages and group 2 innate lymphoid cells (ILC2s), contribute to asthma. This study used mice to investigate how M2 macrophages communicate with ILC2s through tiny particles called extracellular vesicles (EVs). These EVs carry important molecules that can influence other cells. The researchers found that EVs from M2 macrophages help activate ILC2s, which worsens asthma symptoms. They identified a specific molecule, 4930474H06Rik, in these EVs that plays a key role in this process. By reducing the levels of 4930474H06Rik, they were able to decrease asthma-related inflammation in mice. This suggests that targeting this molecule could be a new way to treat asthma. This research could lead to better treatment for people with asthma by focusing on how immune cells communicate.

This summary was initially drafted using artificial intelligence, then revised and fact-checked by the author.

## Introduction

Asthma is an inflammatory airway disease associated with respiratory symptoms, including chest tightness, shortness of breath and cough^1^. The incidence of asthma has increased substantially over several decades, affecting approximately 300 million people worldwide, and the incidence is estimated to increase to 400 million people by 2025^2^. The pathogenesis of asthma is complex and largely driven by T helper 2 (Th2) cells. However, asthma is caused by the interaction between the innate and adaptive immune systems, which work synergistically to promote airway inflammation^3^. The interaction between the most important population of innate immune cells in airway tissues, such as lung M2 macrophages and group 2 innate lymphoid cells (ILC2s), plays a critical role in the initiation, guidance and maintenance of Th2 airway inflammation and, consequently, has gradually attracted increasing attention^4,5^.

Macrophages are the most common immune cells in the airway, and M2 macrophages in asthma models are closely related to the pathophysiology of asthma. Exposure to allergens can activate immune cells to release a variety of cytokines, notably affecting the reprogramming of macrophages into M2. Specifically, M2 macrophages are a subtype of macrophages with anti-inflammatory and tissue repair functions. However, in the asthma environment, the excessive activation of M2 macrophages exacerbates airway inflammatory responses. The adoptive transfer of M2 alveolar macrophages can aggravate airway inflammation, increase airway hyperresponsiveness and mucus secretion, suggesting that they play an important role in the pathogenesis of asthma^6,7^. ILC2s, activated in the airway epithelium, have a crucial role in the development of asthma. In response to various triggers, such as external allergens, viruses and bacteria, cigarette smoke and inflammatory substances, epithelial cells respond by producing ‘alarms’, including IL-33, IL-25 and thymic stromal lymphopoietin. Then, activated ILC2s produce numerous type 2 cytokines, including IL-5 and IL-13, as part of a rapid response to these environmental signals, and induce type 2 cytokines^5^, which in turn promote eosinophilic inflammation and goblet cell metaplasia, which occur independently or in concert with adaptive immunity. In recent years, there has been a growing appreciation for the role of macrophages and ILC2s crosstalk in various diseases^8–10^. However, the pathways related to this crosstalk remain to be further studied.

Macrophages exert their immunomodulatory effects via soluble factors, cell–cell contacts, and extracellular vesicles (EVs)^11,12^. EVs are lipid bilayer membrane-wrapped nanoparticles released by almost all types of cells. EVs perform cell‐to‐cell communication by transferring nucleic acids, proteins and enzymes derived from specific host cells to target recipient cells, and thereby have significant clinical potential^3,13,14^. Recently, M2 macrophage-derived EVs (M2 EVs) were identified as key messengers of intercellular communications related to the invasion and metastasis of tumor cells, inflammatory diseases and immunotherapy^15–19^. However, the mechanism by which M2 EVs regulate ILC2s in allergic airway inflammation is unclear. Given the critical role of ILC2s in both initiating and amplifying allergic airway inflammation, it is crucial to understand the molecular mechanisms of M2 EVs to control the response of ILC2s and thus control allergic airway inflammation.

Here, we demonstrated that EVs from lung M2 macrophages enhanced the response of ILC2s to promote allergic inflammation. Using neutral sphingomyelinase (N-SMase) inhibitor (GW4869)-treated mice, we showed that activation of ILC2s was impaired when the mice were exposed to papain. However, adoptive transfer of M2 EVs restored activation of ILC2s, suggesting that the maintenance of ILC2s activation is at least partially dependent on M2 EVs. On the basis of RNA sequencing results, mouse long non-coding RNA (lncRNA) 4930474H06Rik was selected as a potential mediator of these effects of M2 EVs. Inhibiting the expression of 4930474H06Rik significantly alleviated allergic airway inflammation; therefore 4930474H06Rik is expected to become a potential target for the treatment of allergic airway inflammation.

## Materials and methods

### Mice

C57BL/6 female mice and *Rag1*^*−/−*^ female mice, aged between 6 and 8 weeks and weighing 20–22 g, were sourced from GemPharmatech Co., Ltd. and Cyagen Biosciences Co., Ltd., respectively. The mice were kept in pathogen-free environments. All animal experiments were performed according to the Guide for the Care and Use of Laboratory Animals (Ministry of Health, China, 1998) and the guidelines of the Laboratory Animal Ethical Commission of Wannan Medical College. The experimental protocols were evaluated and approved by the Animal Ethical Committee of Wannan Medical College (approval no. LLSC-2021-136).

### Cell culture

Bone marrow-derived macrophages (BMDMs) were isolated from BALB/c mice following established protocols^20^. Initially, a conditioned medium was created using 20% supernatant from culturing L929 mouse fibroblasts, combined with 20% fetal bovine serum (FBS) and 1% penicillin/streptomycin. Cells extracted from the bone marrow of the femur and tibia of mice were washed in the conditioned medium under sterile conditions and then cultured. On day 3, the culture medium was refreshed, and on day 6, non-adherent cells were discarded; the remaining adherent cells constituted BMDMs (M0). The M0 macrophages were treated with 20 ng/ml IL-4 (PeproTech) in six-well plates (1 × 10^6^ cells/well) to produce M2 macrophages. After 48 h of stimulation, the cells were collected and analyzed using flow cytometry and real-time quantitative PCR (RT–qPCR).

### Mouse model of asthma

The mouse asthma model induced by papain was slightly modified according to a method described in a previous study^21^. Mice aged 6 weeks were sensitized with 200 μg of papain (mixed with aluminum hydroxide) via intraperitoneal (i.p.) injection on days 0 and 7. This was followed with additional papain (100 μg in a volume of 40 μl PBS) via intranasal (i.n.) administration on days 14–17. After the papain treatment was completed, the mice were euthanized and tissue samples were collected for analysis.

### Isolation of EVs from lung tissues

EVs were isolated from lung tissue and BMDMs (M2) through differential ultracentrifugation^20^. In brief, the lung tissue was incubated with 275 U/ml collagenase type II solution for 30 min in a shaking water bath at 37 °C. After filtering the cell suspension through a 100 μm filter, the sample was centrifuged at 3000*g* for 20 min and then at 10,000*g* for 60 min at 4 °C to remove cells, debris and large vesicles. Subsequently, the supernatant was filtered through a 0.22 μm filter and then ultracentrifuged at 110,000*g* for 2 h at 4 °C using a Beckman Coulter Optima XPN-100 ultracentrifuge to collect the EVs. The same centrifugation procedure was also used for collecting M2 EVs from BMDMs.

### Characterization of M2 EVs

Transmission electron microscopy (TEM) was performed to determine the morphology of M2 EVs. The EVs were placed on a copper mesh and allowed to rest for 2 min. Then, filter paper was used to blot the remaining liquid. The EVs were then stained with a 3% phosphotungstic acid solution at room temperature for 5 min and air dried naturally. Finally, the EVs were examined and photographed using TEM.

We used nanoparticle tracking analysis (NTA) to determine the size of the EVs. First, we cleaned the sample wells with deionized water and conducted machine calibration using 100 nm polystyrene microspheres. Once calibration was completed, the EVs were diluted to a suitable concentration for analysis. Finally, we collected and analyzed the results obtained from the NTA measurements.

M2 EVs were also characterized by western blotting. The EVs were lysed using RIPA lysis buffer on ice, and the protein concentration was determined using a BCA protein quantification kit. The EV protein samples underwent separation via SDS–PAGE and were subsequently transferred to PVDF membranes. The membranes were then blocked with 5% skimmed milk at 4 °C for 1 h and incubated overnight at 4 °C with various primary antibodies (anti-CD63, 1:1,000, Abcam; anti-CD81, 1:1,000, Abcam; anti-HSP70, 1:1,000, Abcam; anti-calnexin, 1:1,000, Abcam). After washing the membrane, it was incubated at room temperature for 1 h with horseradish peroxidase-conjugated antibody. Finally, the chemiluminescent signals were analyzed using an automatic chemiluminescence image analysis system (Tanon 5200, Tanon Science & Technology).

### Sorting and cell culture of ILC2s

ILC2s were extracted from the lung tissue of mice using an ILC2s enrichment kit from Stem Cell. The ILC2s were cultured at 37 °C and 5% CO_2_ for 3 days in DMEM containing 100 U/ml penicillin/streptomycin and 10% FBS and supplemented with IL-2 (20 ng/ml), IL-7 (20 ng/ml) and IL-33 (200 ng/ml)^22^. To investigate the functional impact of M2 EVs on ILC2s, M2 EVs at a concentration of 200 µg/ml were infused into the culture medium of ILC2s.

### Internalization mechanism of M2 EVs

To explore the internalization mechanism of M2 EVs, we initially treated the cells with diverse inhibitors: 80 μM dynasore, 10 μM chlorpromazine or 100 μM amiloride. The cells were subsequently incubated with PKH26-labeled M2 EVs for 1 h. The proportion of PKH26-labeled M2 EVs in lung cells was analyzed by flow cytometry.

### Macrophages depletion

To deplete mice of macrophages, 100 µl of clophosome was administered intranasally to the mice once per day for 3 days, followed by injections of 200 µg papain once per day for 4 days. To determine the efficiency of the macrophage clearance, the expression of F4/80 was evaluated using flow cytometry.

### Lung histology and airway mucus expression

Asthmatic mice in each treatment group were injected through the heart with precooled PBS, followed by paraformaldehyde under deep anesthesia. The lungs were then removed and soaked in fresh 4% neutrally buffered paraformaldehyde at room temperature for 24 h. The paraffin-embedded tissues were sliced (7 μm thickness), followed by hematoxylin and eosin (H&E) and periodic acid–Schiff (PAS) staining. The degree of pulmonary inflammatory infiltration was assessed under a microscope by two researchers. H&E staining was performed using the following scoring scale^4^: 0, normal; 1, a few inflammatory cells; 2, a ring of inflammatory cells one cell layer deep; 3, a ring of inflammatory cells 2–4 cells deep; and 4, a ring of inflammatory cells more than four cells deep. Airway mucus secretion was also scored on a scale of 0–4, with 0 assigned to tissue negative for airway mucus secretions and 1–4 assigned to tissue positive for PAS-staining bronchi. The extent of mucus production in positive bronchi was scored as follows^5^: 1, 5–25%; 2, 25–50%; 3, 50–75%; and 4, >75%.

### Flow cytometric analysis

For in vivo experiments, the lung tissue cell suspension was lysed to remove red blood cells and then seeded in 24-well plates. Then, phorbol-12-myristate-13-acetate (MultiSciences) and brefeldin A (MultiSciences) were added to the cell culture medium for stimulation. After 5 h, the cells were collected and blocked with anti-CD16/CD32 (clone 93, Thermo Fisher Scientific). Then they were stained with the following fluorescently labeled monoclonal antibodies at 4 °C for 30 min: anti-CD45 (clone 30-F11, BD Biosciences), anti-lineage (clone 17A2, RA3-6B2, M1/70, Thermo Fisher Scientific), anti-CD90 (clone 53-F2.1 Thermo Fisher Scientific) and anti-ST2 (clone RMST2-2, Thermo Fisher Scientific). Next, intracellular cytokine staining of ILC2s was performed using the eBioscience Intracellular Fixation and Permeabilization Buffer Set (Thermo Fisher Scientific), namely IL-5 (clone TRFK5, Thermo Fisher Scientific) or IL-13 (clone eBio13A, Thermo Fisher Scientific). For M2 EVs co-cultured with ILC2s, stimulated with PMA and brefeldin A after 24 h of co-culture, followed by the same staining protocol.

For Ki67 staining, after surface staining of ILC2s in the lung tissue cell suspension, the True-Nuclear transcription factor buffer kit (BioLegend) was used, followed by incubation with anti-Ki67 (clone SolA15, Thermo Fisher Scientific) antibody for 30 min.

To analyze M1 and M2 macrophages, cell suspensions of lung tissue were first stained with anti-F4/80 (AF13724, Elabscience) for 30 min and then stained with anti-CD86 (AF10959, Elabscience) and anti-CD206 (clone MR6F3, Thermo Fisher Scientific) following the instructions for using eBioscience intracellular fixation and permeabilization buffer reagents. Once the staining procedure was completed, the samples were washed with PBS and then resuspended in PBS to facilitate flow cytometric analysis. To stain eosinophils in lung tissue, we directly labeled them with anti-CD11c (clone N418, Thermo Fisher Scientific) and anti-Siglec-F (clone 1RNM44N, Thermo Fisher Scientific), and then detected the labeled cells using flow cytometry.

### ELISA for cytokine determination

The concentration of Th2-associated cytokines in the bronchoalveolar lavage fluid (BALF) of mice was quantified using enzyme-linked immunosorbent assay (ELISA) kits obtained from MultiSciences Biotech Co., Ltd. All experimental procedures were conducted in accordance with the manufacturer’s guidelines.

### EVs RNA sequencing

M0 and M2 EVs RNA sequencing was performed by RiboBio. Total RNA from EVs was used for the library preparation and sequencing, both of which were conducted by RiboBio. The RNA fragments were approximately 200 bp. The collected RNA was synthesized by first- and second-strand cDNA according to the instructions provided on a NEBNext Ultra kit, followed by transcript screening and low cycle enrichment using an Illumina RNA library preparation kit (NEB). After purification, the library products were evaluated and identified using an Agilent 2200 TapeStation and Qubit 2.0 (Life Sciences), followed by further sequencing using a HiSeq30000 (2× 150 bp). Then, the low-quality reads and those containing poly-N and adapters were removed from the raw data to obtain clean, high-quality reads. The clean reads were aligned with the default parameters of the mouse reference genome using HISAT2. The aligned short reads were then converted into reads for each gene model using HTSeq. The read counts were then used as input to DEseq to evaluate the differential gene expression with the Benjamini Hochberg multiple test correction method enabled. We selected differentially expressed genes using a threshold of |log2 (fold change)| ≥1 and *P* value <0.05. The raw sequence data were deposited in the Genome Sequence Archive (Genomics, Proteomics and Bioinformatics 2021) at the National Genomics Data Center (Nucleic Acids Res 2022), China National Center for Bioinformation/Beijing Institute of Genomics, Chinese Academy of Sciences (GSA: CRA009657), which are publicly accessible at https://ngdc.cncb.ac.cn/gsa.

### Real-time quantitative PCR

Total RNA was extracted from cells using Trizol reagent (Invitrogen), and cDNA was synthesized using a cDNA synthesis kit (Applied Biosystems). Subsequently, RT–qPCR was performed on a Lightcycler 480 system (Roche) with the cDNA serving as a template and using the primers listed in Supplementary Table 1. The cDNA was then further amplified with universal reverse primers and specific forward primers with cycling conditions as follows: predenaturation for 2 min at 95 °C, then 40 cycles of 10 s at 94 °C, 15 s at 58 °C, and 20 s at 72 °C. Subsequently, melting curve analysis was conducted. All reactions were performed in triplicate. The mRNA expression levels were normalized to *GAPDH* and computed using the 2^−ΔΔCt^ method.

### FISH

RNA fluorescence in situ hybridization (FISH) procedures were conducted in accordance with the manufacturer’s instructions (Genepharma) and a previous publication^23^. Briefly, cultured cells were washed twice with PBS, fixed in 4% paraformaldehyde in PBS for 15 min and then washed twice with PBS. The cells were then treated with 0.1% buffer A for 15 min and then washed twice with PBS. The cells were incubated with 2× buffer C at 37 °C for 30 min and then incubated with a hybridization buffer containing a Cy5-labeled 4930474H06RiK probe at 37 °C for 16 h. Following incubation, the cells were washed sequentially with 0.1% buffer F, 2× buffer C and 1× buffer C. The nuclei were stained with 4,6-diamidino-2-phenylindole (DAPI) and washed twice with PBS. Finally, the images were acquired via confocal laser scanning microscopy (CLSM).

### Transfection of the 4930474H06Rik smart silencer in M2 macrophages

The 4930474H06Rik smart silencer targeting 4930474H06Rik, presented in Supplementary Table 2, was synthesized by RiboBio. M2 macrophages were inoculated in six-well plates and transfected with the 4930474H06Rik smart silencer or negative control using transfection reagents (RiboBio). The final concentration was 100 nM. The interference efficiency was determined by RT–qPCR after 48 h.

### ECAR

The extracellular acidification rate (ECAR) of ILC2s was assessed using a Seahorse XFe24 extracellular flux analyzer from Agilent Technologies. ILC2s were co-cultured with either normal control (NC) or smart silencer-treated M2 EVs in an XF 24-well plate at a density of 1 × 10^4^ cells per well, following standard protocols. The ECAR measurements were conducted using a Seahorse XF glycolysis stress test kit (Agilent). Data analysis was performed with Seahorse WAVE software (Agilent), and the results were expressed as mpH/min.

### Statistics

Statistical analyses were conducted using GraphPad Prism 10 software. For comparisons between two groups, *P* values were calculated using an unpaired two-tailed Student’s *t*-test, unless otherwise specified. For comparisons involving more than two groups, two-way analysis of variance (ANOVA) was used. A *P* value less than 0.05 was deemed statistically significant.

## Results

### The EVs secreted by M2 macrophages in lung tissue are involved in activating ILC2s

**Fig. 1:**
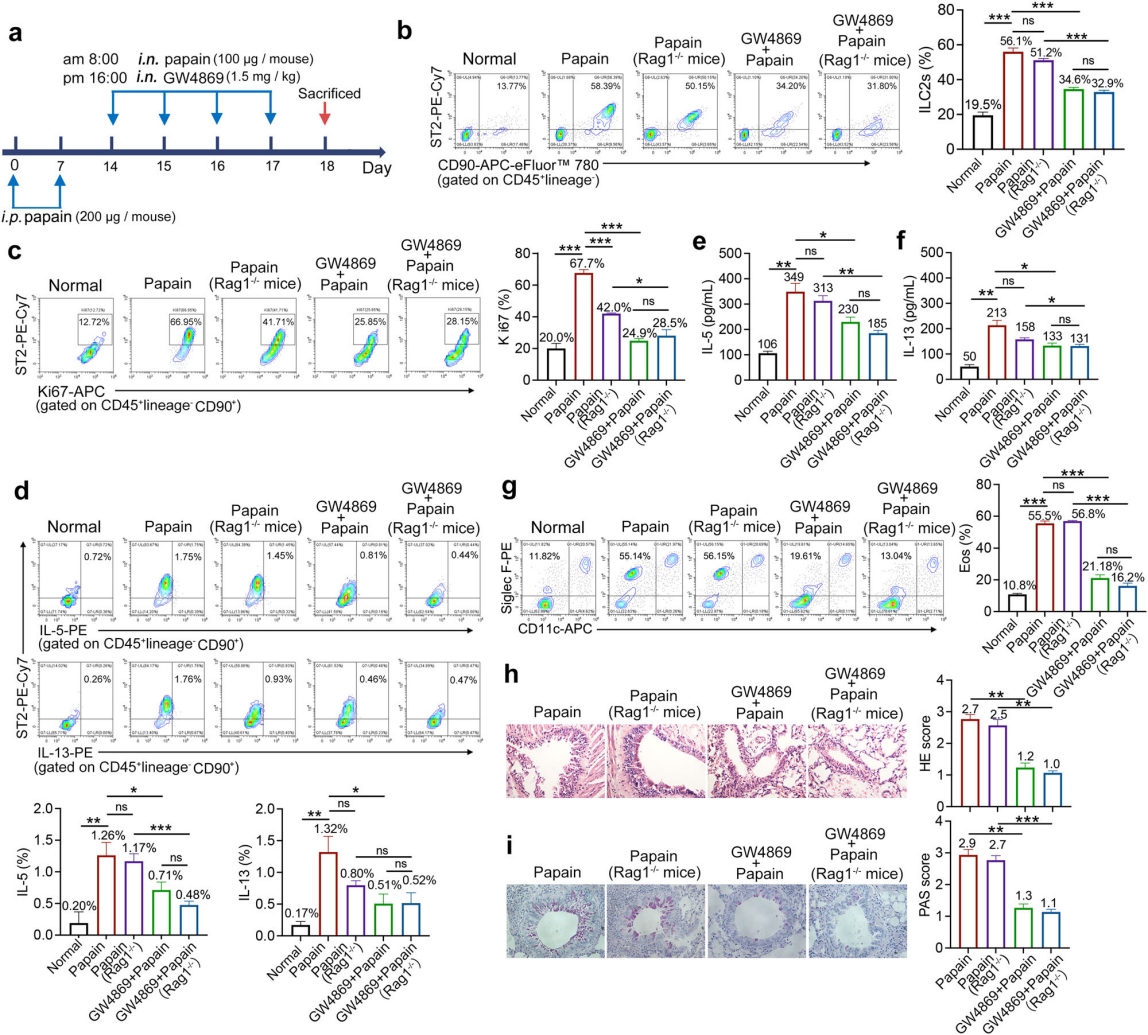
GW4869 injected into asthmatic mice reduced the activation of ILC2s. **a** WT mice and *Rag1*^*−/−*^ mice were sensitized via i.p. injection with 200 μg of papain on day 0 and 7, followed by i.n. administration of papain (100 μg) and GW4869 (1.5 mg/kg) on days 14–17. After the last treatment was completed, the mice were euthanized 24 h later, and samples were collected for analysis. **b** Flow cytometric analysis of lung ILC2s (CD45^+^Lin^−^CD90^+^ST2^+^); *n* = 4 mice. **c** The proliferation of ILC2s was indicated by Ki67 staining; *n* = 4 mice. **d** Flow cytometric analysis of IL-5 and IL-13 in ILC2s; *n* = 4 mice. **e**, **f** IL-5 (**e**) and IL-13 (**f**) concentrations (pg/ml) in BALF were determined by ELISA; *n* = 4 mice. **g** The frequencies of eosinophils (CD11c^−^Siglec F^+^) in BALF were analyzed by flow cytometry; *n* = 4 mice. **h**, **i** Representative H&E staining (**h**) and PAS staining (**i**) of lung tissue sections from the indicated groups; *n* = 4 mice. The data are representative of two or three independent experiments and are presented as mean ± s.e.m. *P* values were calculated using two-sided Student’s *t*-tests or one-way ANOVA, followed by a multiple comparison test. ns not significant, *P < 0.05, **P < 0.01 and ***P < 0.001.

To investigate the impact of lung M2 EVs on ILC2s, we first intranasally injected GW4869 into wild-type (WT) and *Rag*^*−/−*^ mice, with some modifications based on a previous study^24^, as shown in Fig. 1a. Ceramide produced by neutral sphingomyelinase 2 (nSMase2) is crucial for the formation and secretion of exosomes (EVs). GW4869 can inhibit nSMase2, which interferes with membrane bending and budding during EVs formation, thereby reducing the release of EVs from the intracellular environment to the extracellular environment^11,25^. In the in vivo experiments, we found that i.n. administration of GW4869 to WT and *Rag*^*−/−*^ asthmatic mice not only reduced the production of EVs in lung tissue, as evidenced by the decreased expression of CD63 (Supplementary Fig. 1), but also inhibited the expansion (Fig. 1b), proliferation (Fig. 1c) and production of type 2 cytokines (Fig. 1d) induced by papain in ILC2s. Additionally, following GW4869 treatment, there was a notable reduction in the levels of type 2 cytokines present in the BALF of both WT and *Rag*^*−/−*^ asthmatic mice (Fig. 1e, f) and a decrease in the eosinophil accumulation (Fig. 1g). Histological analysis further revealed that GW4869 treatment effectively mitigated airway inflammation (Fig. 1h, i). Importantly, no significant differences were observed in the therapeutic effects between WT and *Rag*^*−/−*^ mice receiving GW4869 as indicated by various parameters, including the ILC2s count, cytokine production levels and pathological alterations within lung tissues. *Rag*^*−/−*^ mice exhibited significant immune deficiencies, particularly in their T and B cell populations. These findings indicated that ILC2s inhibited by GW4869 in *Rag*^*−/−*^ mice remained capable of activation, suggesting that their activation occurred independently of T cell- and B cell-derived EVs.

**Fig. 2:**
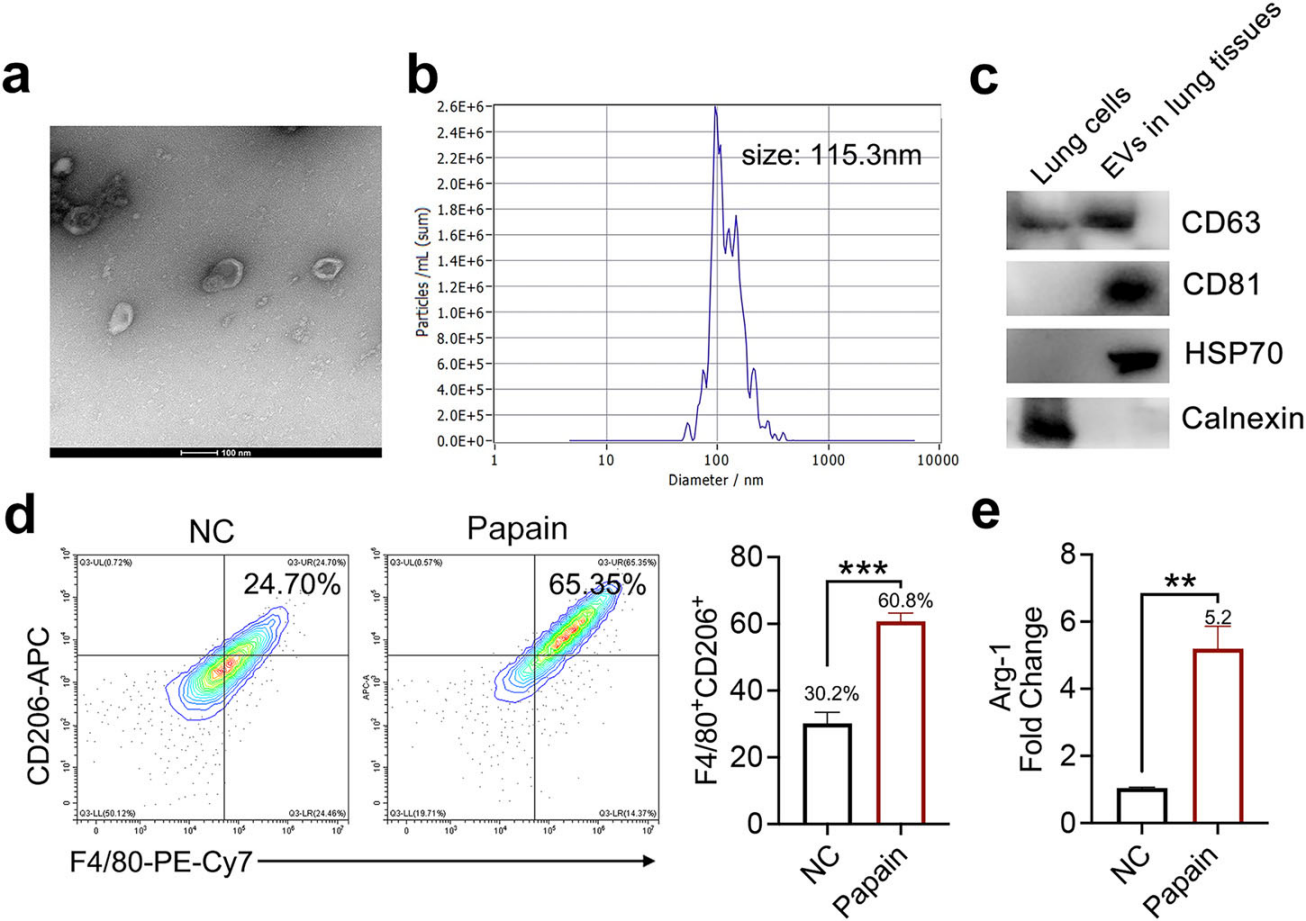
Characterization of lung EVs in asthmatic mice. **a** TEM of lung EVs. **b** Representative results of NTA of lung EVs. **c** Western blot analysis of CD63, CD81, HSP70 and calnexin in EVs from lung tissue. Unedited full blots are shown in Supplementary Fig. 7. **d** The EVs phenotype in the lung tissue of mice treated with PBS (NC mice) and papain was determined by flow cytometry; *n* = 3 mice. **e** The EVs phenotype in the lung tissue of mice treated with PBS (NC mice) and papain was determined by RT–qPCR; *n* = 3 mice. The data are representative of two or three independent experiments and are presented as mean ± s.e.m. *P* values were calculated using two-sided Student’s *t*-tests. **P < 0.01 and ***P < 0.001.

Lung tissue contains a large number of macrophages, which have a crucial role in the immune response and tissue homeostasis^26^ and have been shown to interact closely with ILC2s^10,27^. Therefore, we hypothesized that EVs present in lung tissue may originate from these macrophages. As shown in Fig. 2a, b, both TEM and NTA demonstrated that the isolated EVs exhibited distinct round shapes with consistent sizes, with the largest measuring 115.3 nm. Moreover, western blotting results, as shown in Fig. 2c, indicated that the purified EVs all had higher levels of the EV markers CD63, CD81 and HSP70 when compared with the negative control cell lysate. Importantly, calnexin, which is a marker for the endoplasmic reticulum, was not detected in the EVs. Next, we further examined the components of the EVs to gain a deeper understanding of their properties. Flow cytometry analysis showed that a known marker of M2 macrophage polarization, CD206, was expressed at higher levels in the EVs from the lungs of asthmatic mice compared with those of NC mice (Fig. 2d). Additionally, we evaluated the expression level of another crucial marker of M2 macrophages, arginase 1 (Arg-1), by RT–qPCR (Fig. 2e). The results showed that the expression level of Arg-1 in EVs from the lung tissues of asthmatic mice was significantly higher than that in EVs from the lung tissue of NC mice, further confirming that the lung tissue EVs of the asthmatic mice exhibited an M2 phenotype, which is precisely secreted by M2 macrophages. To summarize, our findings suggested that a significant portion of the EVs present in the lung tissues of asthmatic mice is derived from M2 macrophages and that these EVs are essential for the activation of ILC2s.

### M2 EVs are essential for the enhancement of ILC2s function

**Fig. 3:**
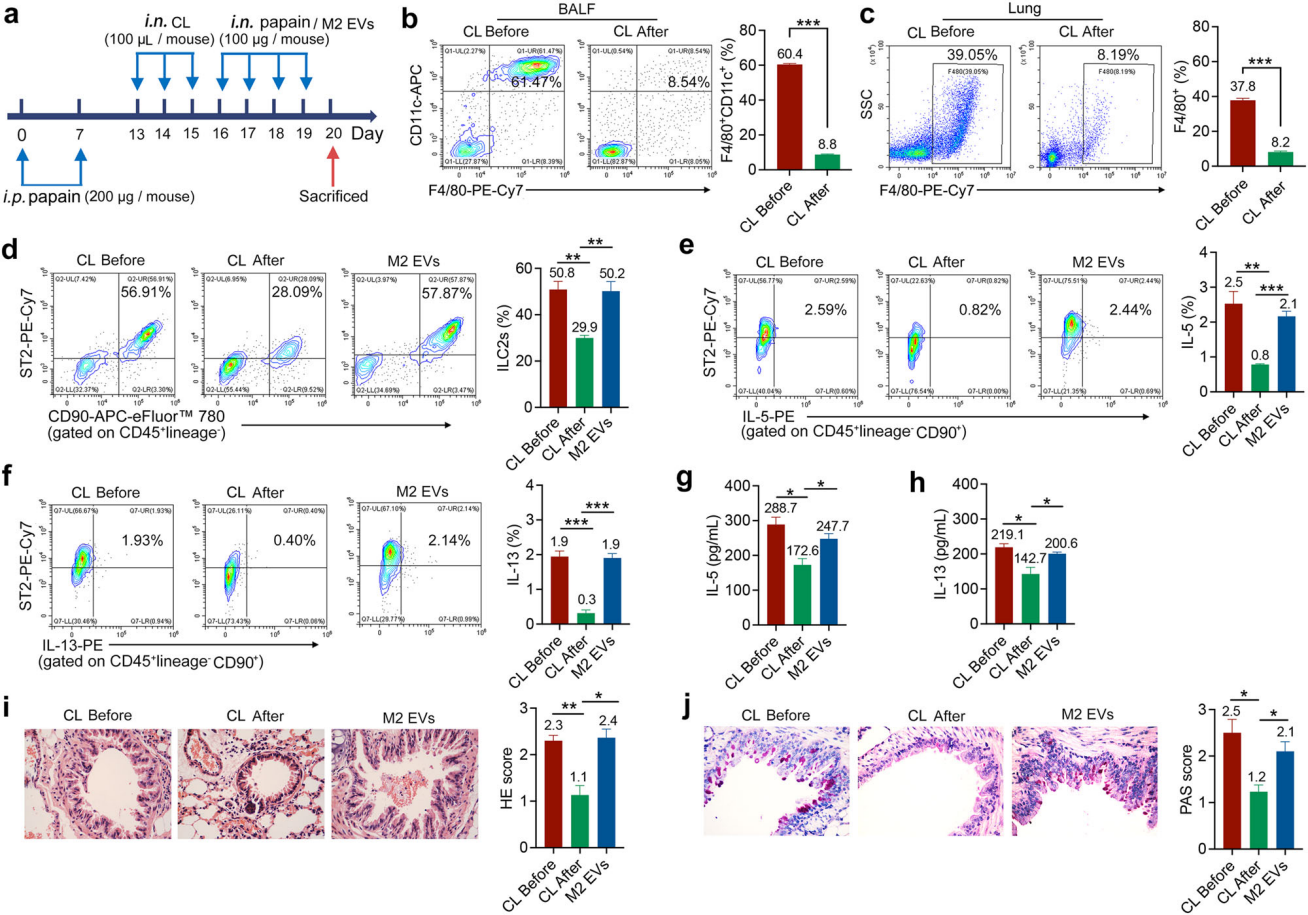
M2 EVs are essential for enhancing ILC2s function. **a** WT mice were sensitized via i.p. injection with 200 μg of papain on day 0 and 7, followed by i.n. administration of CL (100 μl) on days 13–15, and then again with papain (100 μg) intranasally on days 16–19. For the adoptive transfer of M2 EVs, 200 μg of M2 EVs was administered intranasally on days 16–19. After the last treatment was completed, the mice were euthanized 24 h later, and samples were collected for analysis. **b**, **c** Flow cytometry was used to determine the efficiency of macrophages clearance in the BALF (**b**) and lung tissue (**c**) of mice induced by papain; *n* = 4 mice. **d** Flow cytometric analysis of lung ILC2s; *n* = 4 mice. **e**, **f** Flow cytometric analysis of IL-5 (**e**) and IL-13 (**f**) in ILC2s; *n* = 4 mice. **g**, **h** IL-5 (**g**) and IL-13 (**h**) concentrations (pg/ml) in BALF were determined by ELISA; *n* = 4 mice. **i**, **j** Representative H&E (**i**) and PAS (**j**) stained lung sections from the indicated groups; *n* = 4 mice. Data are representative of two or three independent experiments. *P* values were calculated using two-sided Student’s *t*-tests or one-way ANOVA, followed by a multiple comparison test. *P < 0.05, **P < 0.01 and ***P < 0.001.

To further validate the significance of M2 EVs in the activation of ILC2s, we intranasally administered a macrophage-depleting agent (clodronate liposomes, CLs) to eliminate macrophages in mice. The specific steps are detailed in Fig. 3a, which shows the amount and timing of i.n. injections of CLs in the mice. The results of flow cytometry strongly confirmed the significant clearance effect of CLs on the macrophages, with the clearance efficiency exceeding 85% and 78% in the BALF and lung tissue of mice treated with CLs (Fig. 3b, c), respectively. To our surprise, the number of ILC2s decreased significantly after clearing the macrophages (Fig. 3d). This reduction was not only a quantitative change but was also accompanied by a relative decrease in cytokine production associated with key immune responses, including production of IL-5 and IL-13 (Fig. 3e, f). Subsequently, we successfully polarized BMDMs into M2 (Supplementary Fig. 2a, b), and then extracted M2 EVs from the supernatant of M2 macrophages (Supplementary Fig. 2c-e), which were reintroduced into CL-treated mice, thereby restoring the function of ILC2s. These results suggested that macrophages play a key role in maintaining the number and function of ILC2s in tissue. Therefore, understanding the interaction between these two cells is crucial for elucidating the mechanisms of allergic diseases.

Subsequently, we conducted an evaluation of the levels of IL-5 and IL-13 in the BALF of mice. The results demonstrated that the levels of IL-5 and IL-13 in the BALF were significantly lower in the group where macrophages were removed, when compared with the control group. However, these levels were restored following the injection of M2 EVs (Fig. 3g, h). Furthermore, to further substantiate this outcome, a histopathological examination of the lung tissue of mice treated with CLs revealed a decrease in the infiltration of inflammatory cells and a reduction in the mucus secretion by goblet cells, while the degree of lung inflammation increased again after injecting the CLs-treated mice with M2 EVs (Fig. 3i, j). These findings suggested that lung M2 macrophage-derived EVs have a crucial role in maintaining the function of ILC2s. These EVs carry a number of biologically active molecules, including proteins, lipids and RNA species, which can influence cell communication and regulate immune responses.

### ILC2s can internalize M2 EVs in vivo

**Fig. 4:**
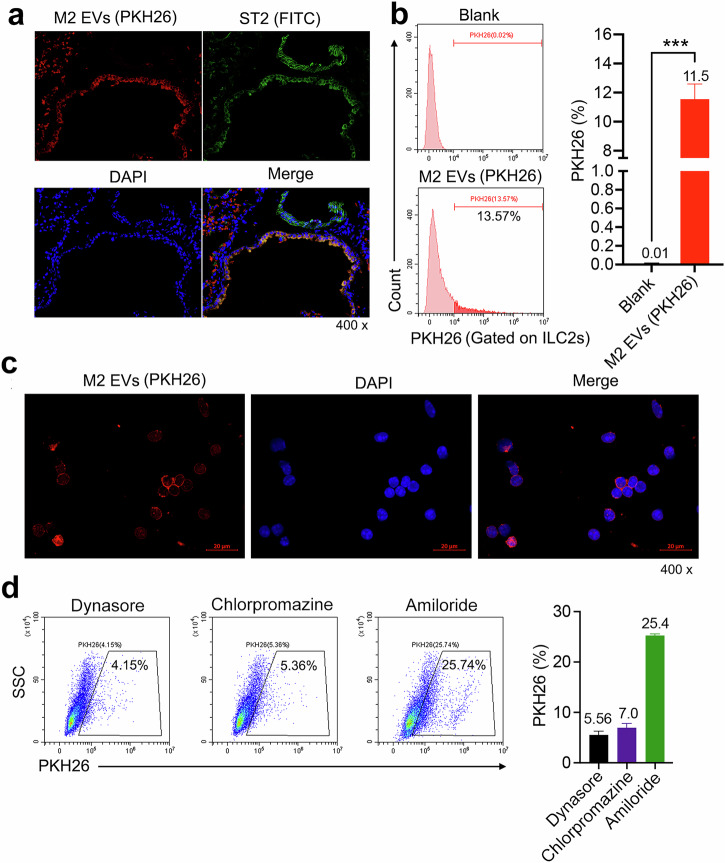
ILC2s internalize M2 EVs. **a** WT mice were sensitized via i.p. injection with 200 μg of papain on day 0 and 7, followed by i.n. administration of papain (100 μg) on days 14–16, and then again with PKH26-labeled M2 EVs (200 μg) intranasally on day 17. The mouse lungs were collected on day 18 and paraffin slices of the lung tissue were generated. Then, ST2 (green, ILC2s marker) was identified by immunofluorescence. DAPI was used as the nuclear stain (blue); *n* = 3 mice. **b** Detection of PKH26-labeled M2 EVs in ILC2s in lung tissue by flow cytometry; *n* = 5 mice. **c** Lung ILC2s were sorted from IL-33-challenged mice and cultured in medium containing IL-2 (20 ng/ml), IL-7 (20 ng/ml) and IL-33 (200 ng/ml) with PKH26-labeled M2 EVs (200 μg/ml) for 24 h. PKH26 was observed in ILC2s by CLSM; *n* = 3 mice. **d** The mechanism of internalization of M2 EVs. Lung ILC2s were sorted from IL-33-challenged mice and then pretreated with different inhibitors (80 μM dynasore, 10 μM chlorpromazine or 100 μM amiloride) for 30 min, followed by treatment with PKH26-labeled M2 EVs for 1 h. Flow cytometry analysis and quantification of PKH26-labeled M2 EVs (red) in ILC2s under different endocytic inhibitors; *n* = 3 wells. The data are representative of two or three independent experiments and are presented as mean ± s.e.m. *P* values were calculated using two-sided Student’s *t*-tests. ***P < 0.001.

To explore whether ILC2s can internalize M2 EVs, we administered PKH26-labeled M2 EVs into asthmatic mice via intratracheal injection. Subsequently, we performed immunofluorescence and flow cytometry analyses on the lung tissue to observe the interaction between ILC2s and the EVs. During the experiment, we used CLSM to acquire images of the lung tissue, which demonstrated co-localization of ILC2s and M2 EVs (Fig. 4a). These results implied that ILC2s were capable of recognizing and interacting with M2 EVs. Additionally, to quantitatively analyze PKH26 signals within the ILC2s population, we performed a detailed flow cytometry analysis (Fig. 4b). The results showed a clearly positive PKH26 signal in ILC2s, further confirming our hypothesis that M2 EVs directly interacted with ILC2s and that ILC2s internalized M2 EVs, which was also confirmed in vitro (Fig. 4c).

Next, we explored the main pathway by which ILC2s internalized M2 EVs by using different inhibitors (Fig. 4d). First, we selected a dynamin inhibitor, namely dynasore^28^. We co-cultured dynasore-pretreated ILC2s with PKH26-labeled M2 EVs to investigate the effects of dynasore on the internalization efficiency of M2 EVs by ILC2s. The results indicated that following dynasore treatment, the internalization capacity of ILC2s for M2 EVs significantly diminished, and approximately 95% of M2 EVs were inhibited from being internalized. This discovery indicated that the mechanism mediated by dynasore is crucial for ILC2s to absorb endogenous EVs. To comprehensively assess other possible mechanisms involved in the internalization process of M2 EVs by ILC2s, we also selected other inhibitors for further investigation. Chlorpromazine, a well-known small-molecule drug that blocks clathrin-mediated endocytosis, was used to reveal the potential contribution of clathrin in this process^29^. We also used amiloride (macromolecule phagocytosis pathway inhibitor) as an inhibitor to disrupt these specific pathways. The results showed that ILC2s showed a diverse uptake capacity when exposed to M2 EVs. This finding suggested that these cells do not rely on a single pathway for EVs uptake, but instead have the flexibility to select different endocytosis mechanisms to adapt to environmental changes. Among all the observed pathways, the dynamic clathrin-mediated endocytosis pathway, the small vesicle pathway and the macromolecule phagocytosis pathway were considered to be the main pathways of EVs uptake. In summary, ILC2s use multiple endocytotic pathways for EVs uptake, providing new insights into their role in immune regulation.

### M2 EVs also indirectly influence ILC2s by mediating macrophages and CD4^+^ T cells

**Fig. 5:**
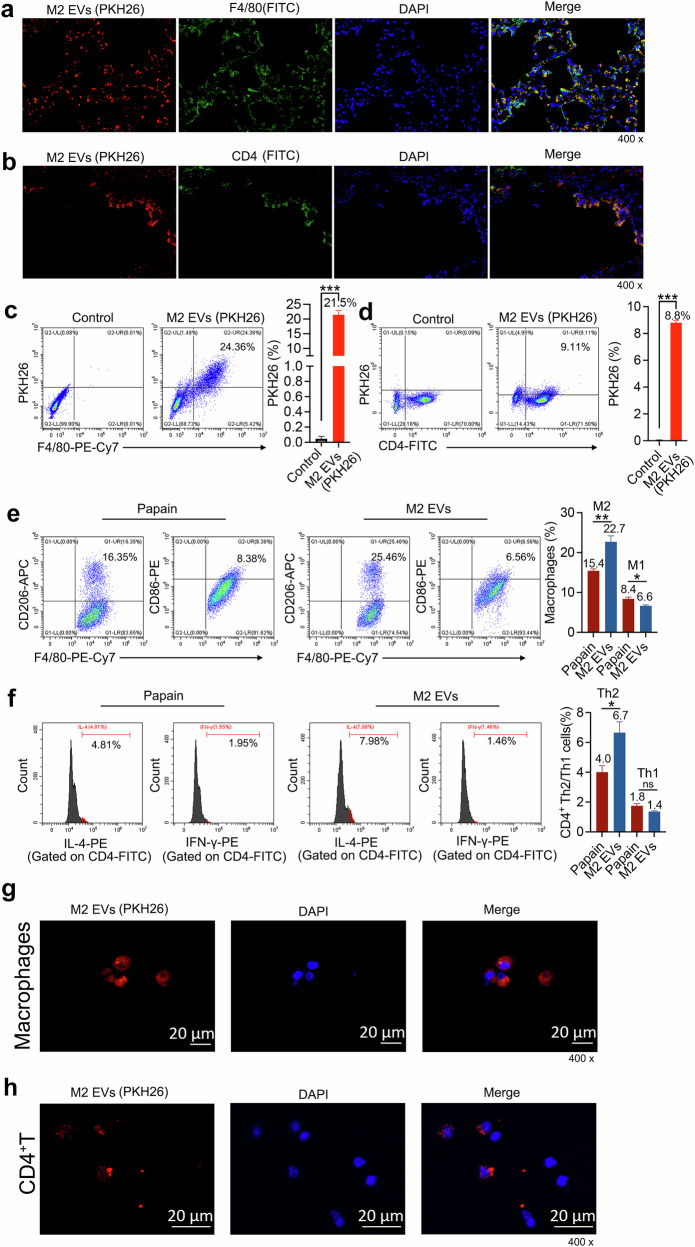
M2 EVs indirectly affect ILC2s through macrophages and CD4^+^ T cells. **a**, **b** WT mice were sensitized via i.p. injection with 200 μg of papain on day 0 and 7, followed by i.n. administration of papain (100 μg) on days 14–16, and then again with PKH26-labeled M2 EVs (200 μg) via the nose on day 17. The mouse lungs were collected and processed into paraffin slices on day 18. Then, F4/80 (green, macrophages marker) (**a**) and CD4 (green, CD4^+^ T cells marker) (**b**) were detected by immunofluorescence. DAPI was used as a nuclear stain (blue); *n* = 3 mice. **c**, Flow cytometry analysis of macrophages (F4/80⁺ cells) in lung tissue to assess uptake of PKH26-labeled M2 EVs; n = 5 mice. **d** Flow cytometry analysis of CD4⁺ T cells in lung tissue to assess uptake of PKH26-labeled M2 EVs; n = 5 mice. **e** Flow cytometry-based detection of M1 vs. M2 macrophage polarization in lung tissue (e.g., CD86⁺ M1 vs. CD206⁺ M2 macrophages); n = 5 mice. **f** Flow cytometry-based detection of Th1 vs. Th2 CD4⁺ T cell subsets in lung tissue (e.g., IFN-γ⁺ Th1 vs. IL-4⁺ Th2 cells); n = 5 mice. **g** Lung tissue was collected from asthmatic mice and prepared as a cell suspension and cultured in six-well plates in DMEM (containing 100 U/ml of penicillin/streptomycin and 10% FBS) at 37 °C and 5% CO_2_ for 24 h. The cells attached to the plate after 24 h were macrophages from the lung tissue. The macrophages were cultured with PKH26-labeled M2 EVs (200 μg/ml) for 24 h, and then PKH26 in the macrophages was analyzed by CLSM; *n* = 3 mice. **h** CD4^+^ T cells were sorted from IL-33-treated mice using an EasySep Release mouse APC positive selection kit (Stem Cell). Briefly, APC-conjugated CD4 antibodies were added to a lung tissue cell suspension. Next, the suspension was incubated with a selection cocktail. Then, RapidSpheres was added to the cell suspension to obtain CD4^+^ T cells according to the manufacturer’s instructions. CD4^+^ T cells and PKH26-labeled M2 EVs (200 μg/ml) were cultured for 24 h, and PKH26 in CD4^+^ T was analyzed by CLSM; *n* = 3 mice. The data are representative of two or three independent experiments and are presented as mean ± s.e.m. *P* values were calculated using two-sided Student’s *t*-tests. ns not significant, *P < 0.05, **P < 0.01 and ***P < 0.001.

Although M2 EVs can directly affect ILC2s, previous results showed that the internalization rate of M2 EVs by ILC2s was only 11.5%. This implies that although M2 EVs contain key signaling molecules necessary to influence ILC2s function in an immune microenvironment, EVs released by macrophages may also exert effects through interactions with other macrophages and other immune cells. Therefore, even if only a small fraction of ILC2s absorb EVs, the remaining EVs can still indirectly regulate ILC2s through other pathways. Therefore, we studied the internalization rate of M2 EVs by macrophages and CD4^+^ T cells (which are closely related to ILC2s) in lung tissue to provide a more comprehensive understanding of how the different cell types coordinate to regulate ILC2s in the local microenvironment. The results of immunofluorescence sections showed that macrophages and CD4^+^ T cells in the lung tissue co-localized with M2 EVs (Fig. 5a, b), with 21.5% of macrophages internalizing M2 EVs and a lower rate of about 9% of CD4^+^ T cells internalizing M2 EVs (Fig. 5c, d).

Further experiments showed that after M2 EVs were administered to asthmatic mice, the number of M2 macrophages and CD4^+^ Th2 cells in the mice increased significantly. This indicated that M2 EVs indirectly affected ILC2s by promoting the polarization of macrophages to the M2 and enhancing the function of ILC2s (Fig. 5e). Additionally, M2 EVs also regulated CD4^+^ Th2 cells to promote the activation of ILC2s (Fig. 5f). To further verify these results, we separated mouse CD4^+^ T cells and macrophages and cultured them with PKH26-labeled M2 EVs for 24 h to observe their internalization. As shown in Fig. 5g, h, both CD4^+^ T cells and macrophages internalized M2 EVs, indicating that M2 EVs directly acted on ILC2s and indirectly promoted the activation of LC2s through macrophages and CD4^+^ T cells.

### lncRNA 4930474H06Rik in M2 EVs was selected as a potential target of ILC2s

**Fig. 6:**
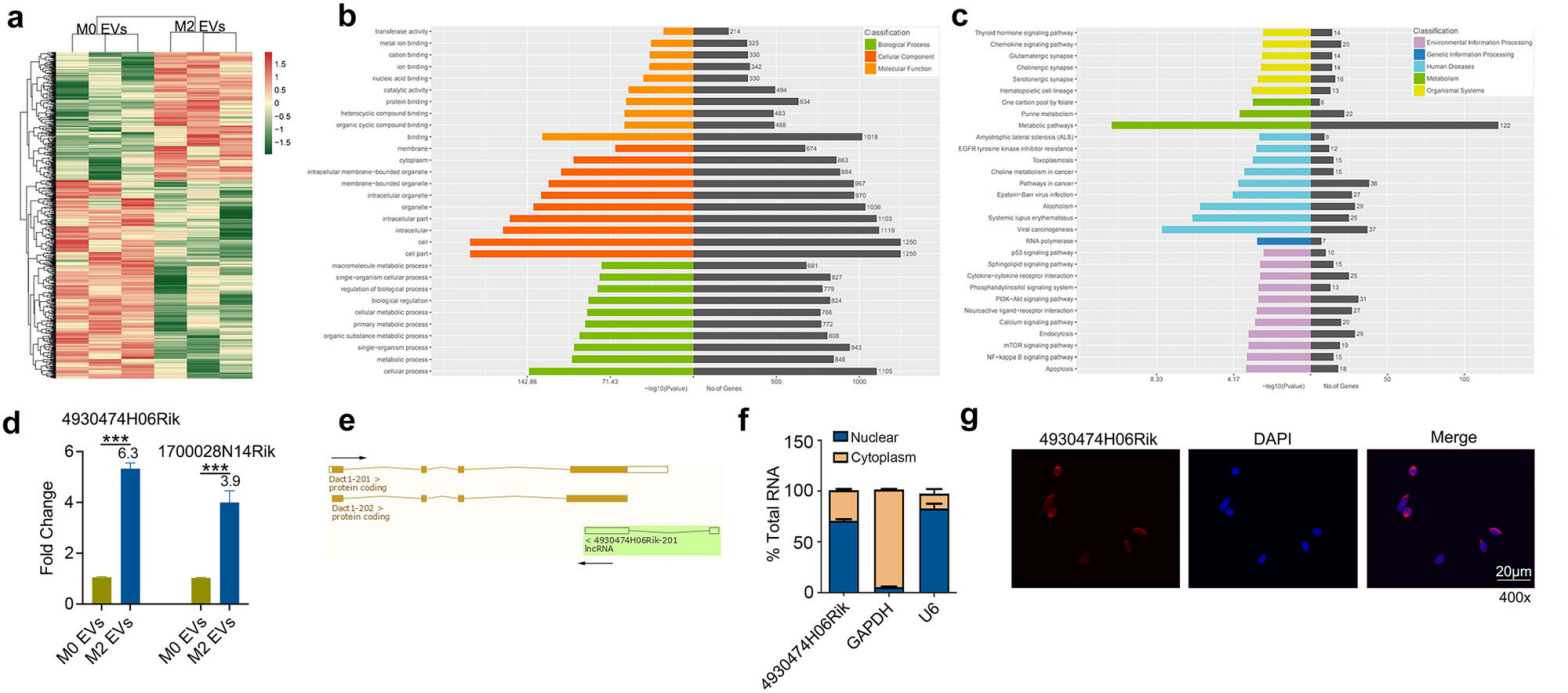
Screening and molecular characterization of lncRNA 4930474H06Rik. **a** A heat map showing the differential expression of lncRNAs between M0 EVs and M2 EVs. **b**, **c** Analyses of the Gene Ontology enrichment (**b**) and Kyoto Encyclopedia of Genes and Genomes pathway enrichment (**c**). **d** Confirmation of the differential expression of lncRNAs by RT–qPCR between M0 EVs and M2 EVs. **e** lncRNA 4930474H06Rik locates on the DACT1 opposite strand. **f** RT–qPCR detection of 4930474H06Rik level in cellular fractions from BMDMs. U6 and GAPDH were the nuclear and cytoplasmic controls, respectively. **g** 4930474H06Rik expression in M2 macrophages were detected by RNA FISH. *P* values were calculated using two-sided Student’s *t*-tests. ***P < 0.001. The data are representative of three independent experiments and are presented as mean ± s.e.m.

In recent years, M2 EVs in different diseases were shown to deliver miRNAs^30,31^. However, although lncRNAs are an important component of EVs, their studies in M2 EVs, especially in asthma, are still limited. Therefore, we urgently need to identify lncRNAs in M2 EVs and explore their role in asthma. We identified 29,577 lncRNAs in both M0 and M2 EVs. A total of 762 lncRNAs were differentially expressed between M0 and M2 EVs, among which 299 lncRNAs were higher in M2 EVs and 463 were higher in M0 EVs (Fig. 6a). Gene Ontology enrichment analyses identified the differentially expressed lncRNAs that were significantly involved in the regulation of cellular components, biological processes, and molecular functions (Fig. 6b). In particular, Kyoto Encyclopedia of Genes and Genomes pathway enrichment analysis revealed that important signaling pathways, including PI3K/AKT, NF-κB and mTOR, were enriched in EVs (Fig. 6c). Previous studies demonstrated that ILC2s proliferation and cytokine generation were associated with these signaling pathways^32,33^. Therefore, the enhanced function of ILC2s may also be closely related to these pathways.

To confirm the RNA sequencing results, two lncRNAs with high expression in the M2 EVs were selected for further confirmation by RT–qPCR. As shown in Fig. 6d, these two lncRNAs were both enriched in M2 EVs. These results were consistent with the microarray data. Next, we selected the lncRNA 4930474H06Rik according to the results of the Gene Importance Calculator (www.cuilab.cn) and gene conservation analysis, for which the homologous gene is NONHSAT037154 (Supplementary Fig. 3). To this end, we evaluated the level of 4930474H06Rik in EVs derived from human macrophages (THP-1 cells). IL-4 and IL-13 stimulation of THP-1 cells induced their transformation into the M2, as evidenced by the morphological changes of THP-1 cells and the expression of Arg-1 in their EVs (Supplementary Fig. 4a, b). Furthermore, 4930474H06Rik was highly expressed in EVs derived from IL-4 and IL-13 polarized THP-1 cells (Supplementary Fig. 4c), suggesting that 4930474H06Rik is highly conserved. Another important reason for selecting 4930474H06Rik was that it is an antisense lncRNA (Fig. 6e), which has a similar effect on its corresponding sense genes due to the complementarity of nucleotide sequences^23^. The gene adjacent to 4930474H06Rik, *DACT1*, was reported to be associated with macrophages and was found to be upregulated in the lung tissue of children with asthma^34^. Our results also demonstrated that, compared with the control group, the expression of 4930474H06Rik in M2 EVs from the lung tissue of asthmatic mice was increased (Supplementary Fig. 5). Therefore, 4930474H06Rik in M2 EVs was selected as a potential target to induce ILC2s activation in allergic airway inflammation.

Then, we further analyzed the molecular characteristics of 4930474H06Rik. The cellular localization of 4930474H06Rik was determined by isolating nuclear RNA and cytoplasmic RNA from M2 macrophages (BMDMs) and measuring the expression of 4930474H06Rik in these two subcellular components. The RT–qPCR data showed that 4930474H06Rik was more highly expressed in the nucleus than in the cytoplasm (Fig. 6f). The standard control U6 RNA was located in the nucleus, whereas GAPDH mRNA was located in the cytosol. RNA FISH experiments also showed that 4930474H06Rik was highly expressed in the nucleus (Fig. 6g).

### M2 EVs-secreted 4930474H06Rik promoted ILC2s activation in vitro

**Fig. 7:**
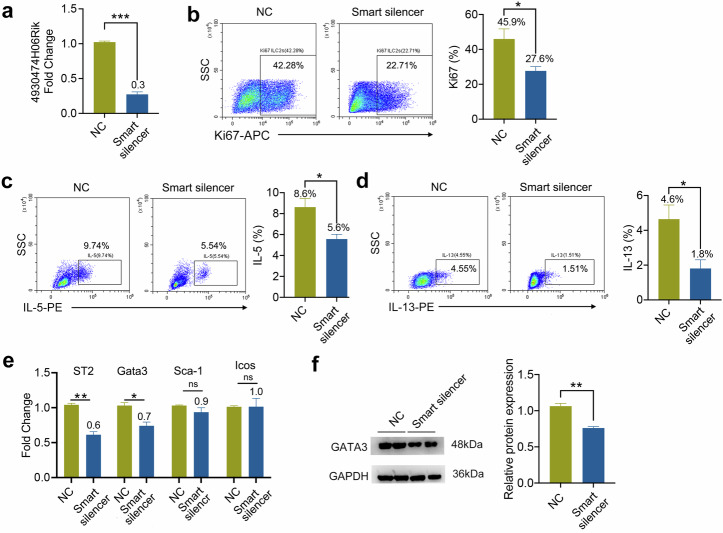
M2 EVs-secreted 4930474H06Rik promoted ILC2s activation in vitro. **a** Knockdown of 4930474H06Rik in M2 macrophages with a smart silencer specifically targeting 4930474H06Rik. Expression of 4930474H06Rik was assessed by RT–qPCR. A nontargeting siRNA negative control (NC) was used; *n* = 3 wells. **b** Lung ILC2s were sorted from IL-33-challenged mice and cultured in medium containing IL-2 (20 ng/ml), IL-7 (20 ng/ml), IL-33 (200 ng/ml), NC or smart silencer-treated M2 EVs (200 μg/ml) for 3 days, and then Ki67 was detected in ILC2s by flow cytometry; *n* = 5 mice. **c**, **d** Flow cytometric analysis of IL-5 (**c**) and IL-13 (**d**) in ILC2s; *n* = 5 mice. **e** The effect of 4930474H06Rik knockdown on the expression of ILC2-related genes analyzed by RT–qPCR; *n* = 5 mice. **f** Western blot analysis of GATA3 expression levels in ILC2s stimulated by NC or smart silencer-treated M2 EVs; *n* = 5 mice. Unedited full blots are shown in Supplementary Fig. 8. The data are representative of two independent experiments and are presented as mean ± s.e.m. *P* values were calculated using two-sided Student’s *t*-tests. ns not significant, *P < 0.05, **P < 0.01 and ***P < 0.001.

We next determined whether 4930474H06RiK promoted ILC2s activation in vitro. To this end, we decreased its expression using a 4930474H06RiK smart silencer in M2 macrophages and evaluated the effects. When the expression of 4930474H06RiK was downregulated, the expression of 4930474H06RiK decreased in M2 EVs (Fig. 7a). Next, we examined the effect of 4930474H06RiK inhibition on the proliferation of ILC2s. After treatment with the 4930474H06RiK smart silencer, the expression of Ki67 in lung ILC2s was reduced (Fig. 7b). The secretion of IL-5 and IL-13 in ILC2s also decreased (Fig. 7c, d). We then used RT–qPCR to examine genes associated with ILC2s activation, including ST2, GATA3, stem cell antigen-1 (Sca-1) and inducible T cell o-stimulator (ICOS)^35–38^. The results showed that 4930474H06RiK inhibition significantly reduced the expression of ST2 and GATA3 but not of Sca-1 or ICOS (Fig. 7e). We further examined changes in the GATA3 protein level via western blotting. The results demonstrated that the expression of GATA3 protein decreased subsequent to 4930474H06RiK inhibition (Fig. 7f). Downregulation of 4930474H06Rik expression in macrophages may lead to changes in the composition of M2 EVs and subsequently weaken the function of ILC2s. To verify this hypothesis, we detected the expression changes of Arg-1 (a gene associated with M2 macrophages) and iNOS (a gene associated with M1 macrophages) in EVs after downregulation of 4930474H06Rik expression by RT–qPCR. As shown in Supplementary Fig. 6, the phenotype of EVs changed from M2 to M1 after downregulation of 4930474H06Rik, which is similar to the previous research results that M1 macrophages can inhibit the expression of ILC2s^39^. Collectively, these results strongly suggested that 4930474H06RiK in M2 EVs regulated ILC2s activation and may be a potential target for treating ILC2s activation in asthma.

**Fig. 8:**
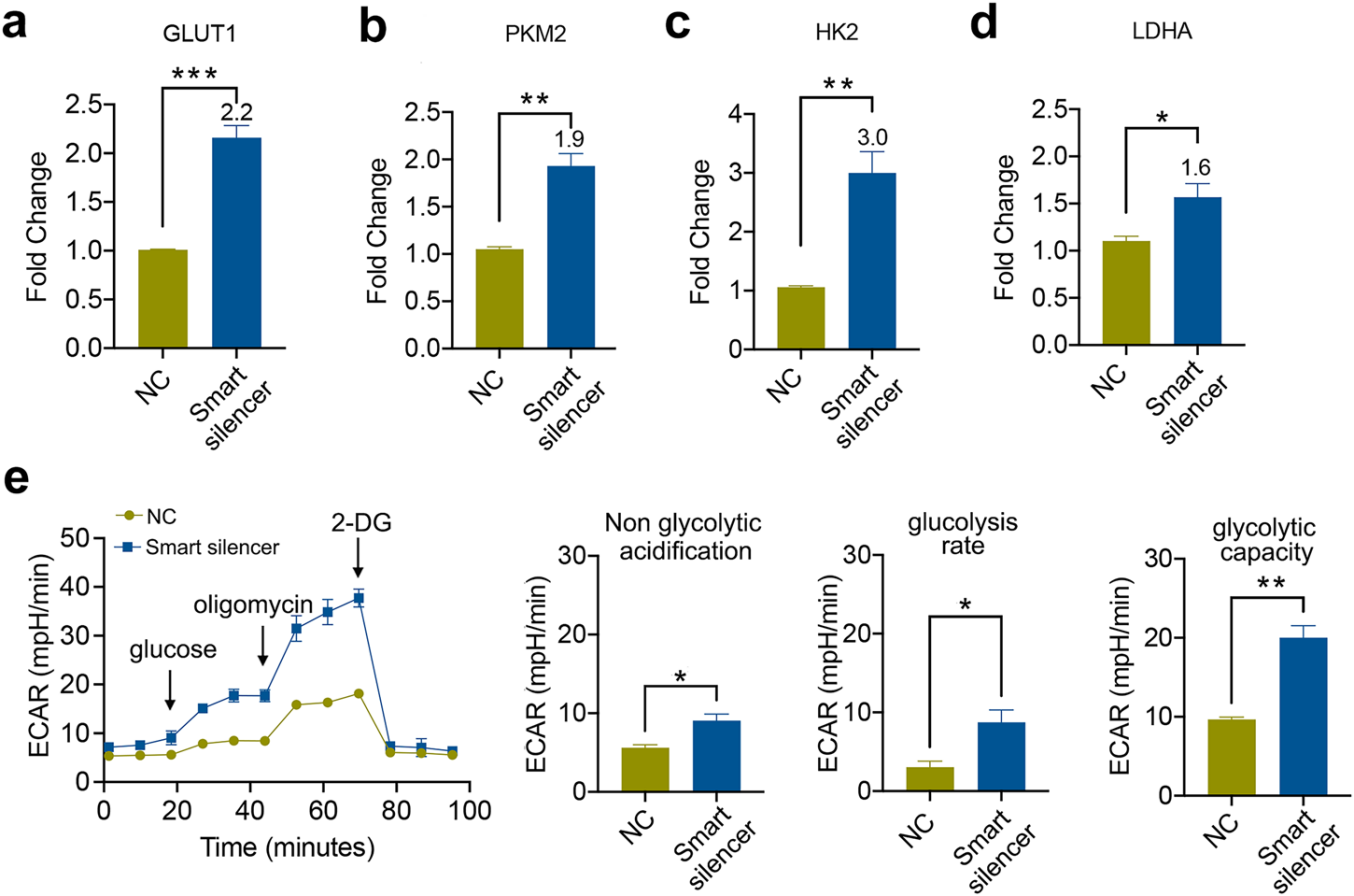
Inhibition of 4930474H06Rik enhanced glucose metabolism in ILC2s. **a**–**d** RT–qPCR analysis and quantification of GLUT1 (**a**), PKM2 (**b**), HK2 (**c**) and LDHA (**d**) in ILC2s stimulated by NC or smart silencer-treated M2 EVs; *n* = 3 wells. **e** The ECAR was measured using a XF96 Seahorse analyzer, of which the glycolysis parameters were quantified; *n* = 3 wells. The data are representative of two or three independent experiments and are presented as mean ± s.e.m. *P* values were calculated using two-sided Student’s *t*-tests. *P < 0.05, **P < 0.01 and ***P < 0.001.

Many studies have indicated that changes in cellular metabolism affect the function of ILC2s^40,41^. Consequently, we aimed to explore whether 4930474H06RiK exerts an impact on the activation of ILC2s by regulating cellular metabolism. ILC2s were isolated from normal mice and co-cultured with M2 EVs or M2 EVs that had been treated with the 4930474H06RiK smart silencer. Subsequently, we investigated the expression levels of four crucial enzymes involved in glycolysis regulation: glucose transporter 1 (GLUT1), hexokinase 2 (HK2), pyruvate kinase M2 (PKM2) and lactate dehydrogenase A (LDHA), as previously reported^42^. The outcomes demonstrated that the expression of these four enzymes in ILC2s co-cultured with M2 EVs treated with the 4930474H06RiK smart silencer was remarkably higher compared with that in the negative control (NC) group, as depicted in Fig. 8a–d. We further used a Seahorse analyzer to study the state of cell glycolysis in ILC2s and observed that the glycolytic rate and glycolytic capacity of ILC2s treated with the smart silencer significantly increased (Fig. 8e). Studies have shown that glycolysis is a potential mechanism of M1-like polarization^42^, and our results suggest that the change of EVs properties induced by 4930474H06Rik downregulation may also lead to the change in the ILC2s phenotype, which leads to the relative increase in glycolysis level.

### M2 EVs-secreted 4930474H06Rik promoted ILC2s activation in vivo

**Fig. 9:**
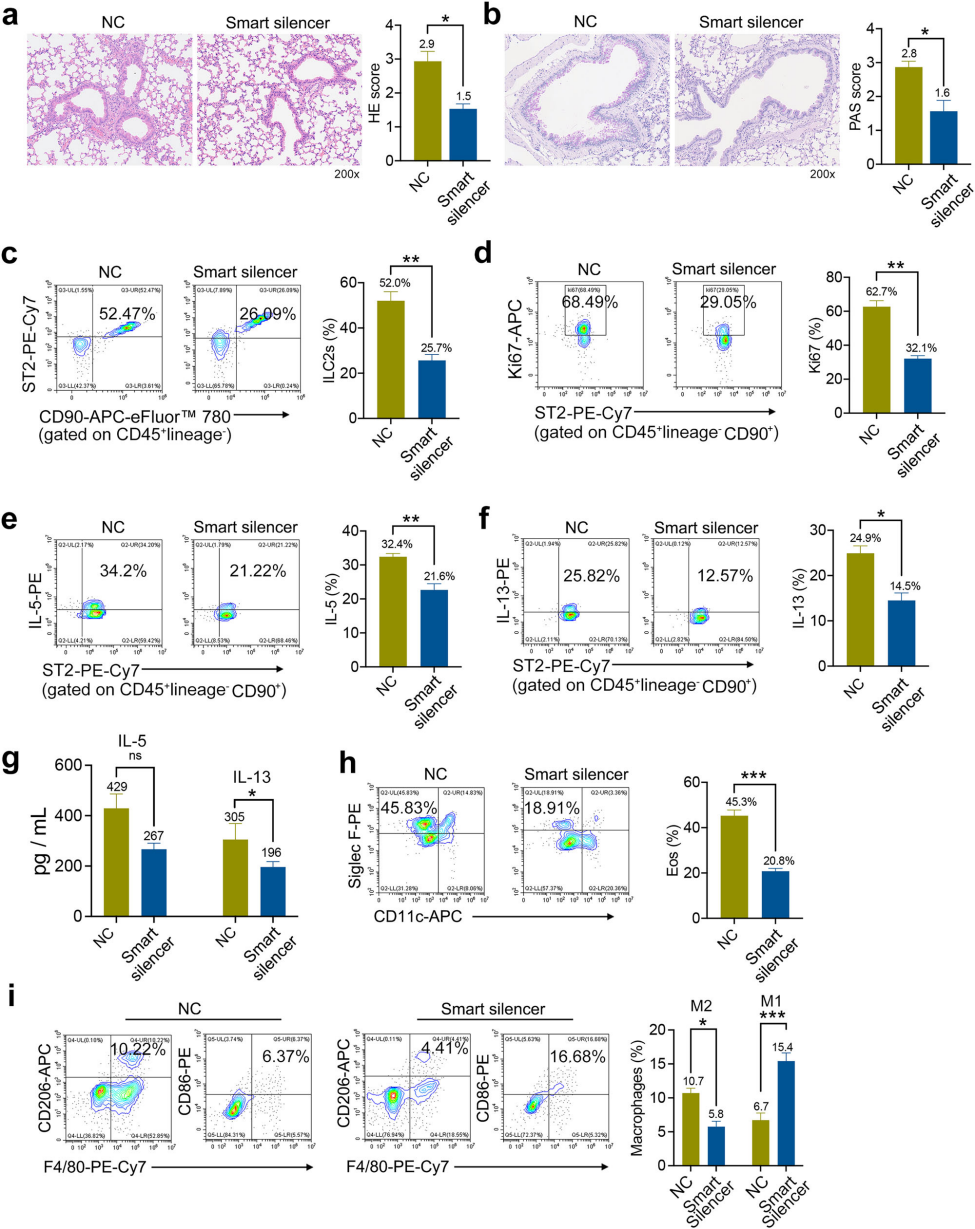
M2 EV-secreted 4930474H06Rik promoted ILC2s activation in vivo. **a**, **b** H&E staining (**a**) and PAS staining (**b**) for mouse lung tissues; *n* = 5 mice per group). **c** The effects of M2 EVs treated with NC and M2 EVs treated with smart silencer on the expression of ILC2s in lung tissue of asthmatic mice were analyzed by flow cytometry; *n* = 5 mice. **d** The percentage of ki67-positive ILC2s in lung tissues was analyzed; *n* = 5 mice. **e**, **f** The percentage of IL-5-positive (**e**) or IL-13-positive (**f**) ILC2s in lung tissues was analyzed; *n* = 5 mice. **g** BALF samples were collected, which were used for measuring the level of the selected cytokine as indicated by ELISA; *n* = 5 mice per group. **h** The frequencies of eosinophils (CD11c^−^Siglec F^+^) in lung tissues were analyzed by flow cytometry; *n* = 5 mice. **i** Detection of M1/M2 macrophages in lung tissue by flow cytometry; *n* = 5 mice. The data are representative of two independent experiments and are presented as mean ± s.e.m. ns, not significant, *P < 0.05, **P < 0.01 and ***P < 0.001 analyzed by two-tailed Student’s *t*-tests for single comparisons.

To further examine the effect of 4930474H06RiK on ILC2s activation in vivo, we established an asthma model using similar modeling approaches as described previously. Mice were administered with NC or 4930474H06RiK smart silencer-treated M2 EVs on day 14, and lung tissue samples were obtained 3 days after i.n. administration of papain. The results showed that compared with NC, those treated with smart silencer showed reduced inflammatory cell infiltration and epithelial goblet cell hyperplasia around the bronchus, concomitant with significantly reduced 4930474H06RiK levels in lung tissue (Fig. 9a, b). We then examined the effects of 4930474H06RiK on the function of ILC2s in lung tissues. Similar to our above findings, we observed that the effects of smart silencer on the levels of ILC2s in lung tissues were mitigated compared with NC (Fig. 9c), accompanied by a decrease in the proliferation level of ILC2s (Fig. 9d). More importantly, the smart silencer scramble significantly decreased the high levels of IL-5 and IL-13 in ILC2s after treatment (Fig. 9e, f), including IL-13 in BALF (Fig. 9g).

Next, to evaluate whether 4930474H06RiK directly influences the severity of papain-induced pulmonary inflammation, the data showed a reduction in the number of eosinophils (Fig. 9h). We also observed that in the lung tissues of mice treated with smart silencer, the number of M2 macrophages decreased while that of M1 macrophages increased (Fig. 9i). Collectively, these data indicate that M2 EVs aggravated allergic airway inflammation in mice, which was partially attributed to the delivery of 4930474H06RiK in M2 EVs to ILC2s.

## Discussion

In this study, we first confirmed that M2 EVs in lung tissue were involved in activating ILC2s in a papain-induced asthmatic mouse model. Subsequently, we established a macrophage depletion model to reveal the importance of lung M2 EVs in the activation of ILC2s. Our study further demonstrated that M2 EVs directly acted on ILC2s and indirectly affected macrophages and CD4^+^ T cells, which in turn enhance the function of ILC2s, exacerbating lung inflammation. We discovered that 4930474H06RiK is highly expressed in M2 EVs and modulates the function of ILC2s through glucose metabolism.

M2 macrophages have an important role in the immune system and are widely reported to promote the progression of various diseases through different mechanisms. These mechanisms include expressing high levels of cellular molecules, secreting soluble factors and transmitting functional molecules via EVs. Specifically, M2 macrophages produce a series of pro-inflammatory and anti-inflammatory factors, and the balance among these factors is crucial for maintaining the health of the body. In our previous studies, we fully demonstrated that deleting key molecules with high expression in M2 macrophages effectively regulated macrophage polarization, providing a new strategy for treating allergic airway inflammation. After further in-depth research, we showed, using in vitro experiments, that co-culturing M2 macrophages with ILC2s significantly inhibited the differentiation process of ILC2s when treated with GW4869 (ref. ^43^). This discovery suggested that M2 macrophages may exert their influence on ILC2s through the EVs pathway. Therefore, in this study, we demonstrated how M2 EVs activated ILC2s in asthma.

Asthma is characterized by airway inflammation, airway hyperreactivity and mucus hyperproduction, and is driven primarily by Th2 cells and the cytokines they release. Increasing evidence suggests that ILC2s are activated by macrophages, such as group V phospholipase A2 (Pla2g5)^10^, to orchestrate the pathological features of pulmonary inflammation, and ILC2s may play a more important role than Th2 cells. However, the effects of M2 EVs on ILC2 activation in asthma or pulmonary inflammation were not reported before this study. The role of EVs in allergic asthma has been reported, in which EVs derived from the airway epithelium promoted allergic airway inflammation^44,45^. Macrophages are the most abundant group of immune cells in lung tissue. They play a very important role, and many recent asthma studies focused strictly on macrophages. Instead, we innovatively investigated the effect of M2 EVs on ILC2s in allergic airway inflammation. We established PKH26-labeled M2 EVs to investigate the internalization of M2 EVs by ILC2s in vivo. We found that M2 EVs were internalized by lung ILC2s in asthmatic mice, but the internalization rate was not particularly high, which led us to speculate that the EVs secreted by macrophages might be internalized by neighboring macrophages or other cells, because the powerful internalization function of macrophages is undeniable. Our data further revealed that M2 EVs were highly internalized by macrophages in vivo, and we found that CD4^+^ T cells also internalized M2 EVs. With an increased proportion of M2 macrophages and CD4^+^ Th2 cells in asthmatic mice, we concluded that M2 EVs directly acted on ILC2s and may indirectly act on ILC2s through M2 macrophages and CD4^+^ Th2 cells. Together, our data extended the current knowledge of M2 EVs in allergic airway inflammation.

EVs contain active components, including proteins, lipids and RNA, which can affect the behavior of adjacent cells^14,15^. There have been many reports on the role of miRNAs in asthma. Research has indicated that altered levels of immune-related miRNAs (such as miR-146a and miR-106b) and inflammatory cytokines (including IL-5 and IL-13) in pediatric asthma may contribute to the progression of the disease^46^. Additionally, Wang et al. found that the dysregulated miR-451a-ETS1 pathway was a distinct molecular mechanism contributing to the onset of childhood asthma^47^. Although the study of miRNAs in EVs is growing, in recent years researchers have become increasingly interested in the role of lncRNAs found in EVs and their potential applications. Research has shown that lncRNAs are an important component of EVs and can significantly affect the function of target cells. Early studies found that lncRNAs, including AGAP2-AS1 (ref. ^48^) and MSTRG.1634.7 (ref. ^49^) from macrophage-derived EVs, participated in immune regulation. Investigating how M2 EVs interact with various immune cells such as ILC2s may offer valuable insights into their potential therapeutic uses. More importantly, through RNA sequencing, we found that 4930474H06RiK was highly expressed in the lung tissue of M2 EVs and asthmatic mice, suggesting that 4930474H06RiK is a potential therapeutic target for asthma.

Mechanistically, our results suggest that the effect of 4930474H06RiK on increasing cytokine secretion and the cell proliferation capacity of ILC2s is achieved by downregulating glycolytic related molecules such as HK2 and so on. This is similar to the research findings of Omid Akbari^32,50^. Nevertheless, some differences cannot be ignored. Previous studies have shown that IL-33 significantly upregulates glycolytis-related genes, especially HIF-1α, in mouse lung ILC2s, which is manifested by increased lactic acid accumulation in ILC2s^51^. These differences may be due to the different metabolic preferences of different cell microenvironments. ILC2s and M2 macrophages mainly rely on lipid utilization to provide energy for cell proliferation and IL-4 and IL-13 secretion, while M1 macrophages are more inclined to use glucose metabolism to enhance cell function^42,52^. Our previous research results also indicate that inhibiting the expression of 4930474H06RiK in M2 EVs also altered the properties of EVs, endowing them with the markers of M1 macrophages. Therefore, it may make a considerable contribution to the phenotypic transformation of ILC2s, and the possibility of ILC2s transforming into type 1 innate lymphoid cells cannot be ruled out. This combined evidence suggests that metabolic alterations occur within ILC2s, and that a lack of 4930474H06RiK increases the level of glucose metabolism in ILC2s.

Certain limitations of the study have not been addressed. First, although we identified the key components in M2 EVs, we have not yet performed in vivo gene editing experiments in macrophages to further clarify the intercellular communication mediated by EVs abnormalities. In the future, it may be necessary to use specific 4930474H06RiK knockout mice or design a targeted inhibitor delivery system for 4930474H06RiK to produce more precise interventions. In addition, the focus of this study was to clarify the effects of M2 EVs on ILC2s. However, the additional mechanisms of action of 4930474H06RiK in EVs have not been elucidated. In subsequent studies, we will examine the mechanisms of action of 4930474H06RiK, including identifying interacting proteins and associated downstream signaling pathways.

## Supplementary information

Supplementary information

## Data Availability

Data will be made available on request.
