# Supplementary material for: Extracellular vesicles derived from lung M2 macrophages enhance group 2 innate lymphoid cells function in allergic airway inflammation
Source: Exp Mol Med. 2025 Jun 2;57(6):1202–15. doi: 10.1038/s12276-025-01465-6 (PMC12229531; doi:10.1038/s12276-025-01465-6)

Extracellular vesicles derived from lung M2 macrophages enhance group 2 innate lymphoid cells function in allergic airway inflammation

Kun Lv[1,2,4+], Yingying Zhang[3+], Guoquan Yin[1,2,5+], Xueqin Li[1,2,4], Min Zhong[1,2,4], Xiaolong Zhu[1,2,4#] and Weiya Pei[1,2,4#]

[1] Central Laboratory, The first affiliated hospital of Wannan Medical College, Wuhu, PR China; [2] Anhui Province Key Laboratory of Non-coding RNA Basic and Clinical Transformation (Wannan Medical College), Wuhu, PR China; [3] Department of Laboratory Medicine, The first affiliated hospital of Wannan Medical College, Wuhu, PR China; [4] Clinical Research Center for Critical Respiratory Medicine of Anhui Province, Wuhu, PR China; [5] Yangzhou Blood Center, Yangzhou, PR China.

[*] **Address correspondence to:** Weiya Pei, Central Laboratory of the first affiliated hospital, Wannan Medical College, 2 Zheshan western road, Wuhu, 241001, PR China.

Tel: +86-553-5739912; **Fax:** +86-553-5739209; **Email:** pwy730@126.com

Running title: M2 macrophages-derived extracellular vesicles promote ILC2s activation in allergic airway inflammation

[+]**These authors contributed equally to this work.**

***Corresponding author:**

Weiya Pei - Central Laboratory, The First Affiliated hospital of Wannan Medical College, Wuhu, PR China. Email: pwy730@126.com.

Xiaolong Zhu - Central Laboratory, The First Affiliated hospital of Wannan Medical College, Wuhu, PR China. Email: zhuxlon3@126.com.

Supplementary tables and figures

Supplementary Table 1. Primers used for qPCR

**List of oligonucleotide sequences**	**5'>3'**
GAPDH (human)-F	ACCATCTTCCAGGAGCGAGAT
GAPDH (human)-R	GGGCAGAGATGATGACCCTTT
GAPDH (mouse)-F	AGGTCGGTGTGAACGGATTTG
GAPDH (mouse)-R	TGTAGACCATGTAGTTGAGGTCA
4930474H06RiK (mouse)-F	GGAGAAGCCTGGCAGCATTCAC
4930474H06RiK (mouse)-R	AGACCCGCACCATTCCAAGTTTAC
4930474H06RiK (human)-F	CCACTAACGATGGGGTGGAC
4930474H06RiK (human)-R	GGGTGCTGTTAGGAGCTGTT
GATA3 (mouse)-F	TCTGGAGGAGGAACGCTAATGGG
GATA3 (mouse)-R	CGGGTCTGGATGCCTTCTTTCTTC
GLUT1 (mouse)-F	AAGAAGAGGGTCGGCAGATGATG
GLUT1 (mouse)-R	AGCACCACAGCGATGAGGATG
LDHA (mouse)-F	CGGCTGGGTCCTGGGAGAAC
LDHA(mouse)-R	ACCTCCTTCCACTGCTCCTTGTC
HK2 (mouse)-F	TGATCGCCTGCTTATTCACGG
HK2(mouse)-R	AACCGCCTAGAAATCTCCAGA
PKM2 (mouse)-F	GGGCCATAATCGTCCTCACC
PKM2 (mouse)-R	TTGCACAGCACAGGGAAGAT
CD206 (mouse)-F	GGAGTGGCAGGTGGCTTAT
CD206 (mouse)-R	TGGACATTTGGGTTCAGGAG
Arg-1(mouse)-R	CGACATCAAAGCTCAGGTGAATCGG

Arg-1 (human)-F	TCTTCTTGACTTCTGCCACCTTGC
ST2 (mouse)-F	GGCACACCATAAGGCTGAGAAGG
ST2 (mouse)-R	CTCCAGAACAGAGCAACCTCAATCC
Sca-1 (mouse)-F	TCCCATTTGAGACTTCTTGCCCATC
Sca-1 (mouse)-R	CCACAATAACTGCTGCCTCCTGAG
ICOS (mouse)-F	GAAGTCCTCTGCGAACTCACCAAG
ICOS (mouse)-R	CTGGGAGCTGTCTGGGTTGTTTAG

Supplementary Table 2. Oligonucleotides sequences used in lncRNA 4930474H06RiK are written.

Genes	Target sequences
4930474H06RiK(Smart silencer mix)	GAAGCCTGGCAGCATTCACG(ASO)<br>CAAAGACAGTGACGTCAACT(ASO)<br>GGACAAAGCATCCAAGTGCT (ASO)<br>GAAGTGGGACATCTGTCAT(siRNA)<br>TCCAAGTGCTTGAGACTGT(siRNA)<br>GGTCTTGAACCCTCAAAGA(siRNA)

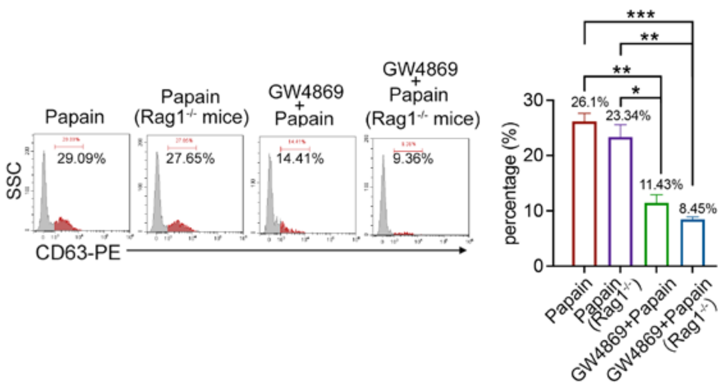

Supplementary Fig. 1. GW4869 inhibited the production of EVs in lung tissue. The exprssion of CD63 in EVs from lung tissue of mice was determined by flow cytometry; n=3 mice. The data are representative of two independent experiments and are presented as mean ± SEM.*P< 0.05, ** P< 0.01, and ***P< 0.001 analysed by two-tailed Student's *t* test for single comparison.

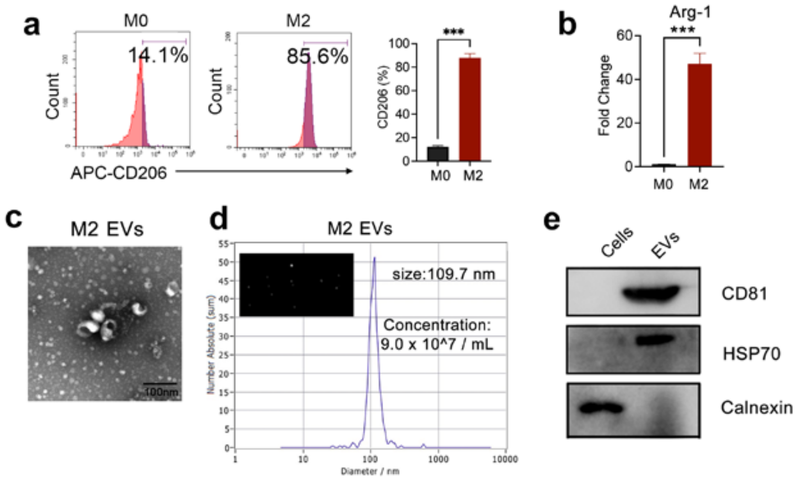

Supplementary Fig. 2. Verification of M2 macrophages and M2 EVs from IL-4 treated BMDMs. (**a**) Flow cytometry analysis of M2 macrophages (BMDMs); (**b**) Arg-1 expression in macrophages (BMDMs) were determined by RT-qPCR. (**c**) TEM of M2 EVs. (**d**) Representative results of nanoparticle tracking analyses (NTA) of M2 EVs. (**e**) Western blot analysis of CD81, HSP70 and Calnexin of M2 EVs from lung tissues. Unedited full blots were in Supplementary Fig. 9. The data are representative of two independent experiments and are presented as mean ± SEM. ***P< 0.001 analysed by two-tailed Student's *t* test for single comparison.

a

Sequence name (Numbered)	Sequence length ▲▼	GIC score ▲▼	Importance ranking (‰) ▲▼
1-ENSMUST00000132822.1	1590	0.684611715781	12
2-ENSMUST00000139621.1	747	0.76341050461	2
Sequence name (Numbered)	Sequence length	GIC score	Importance ranking (‰)

b

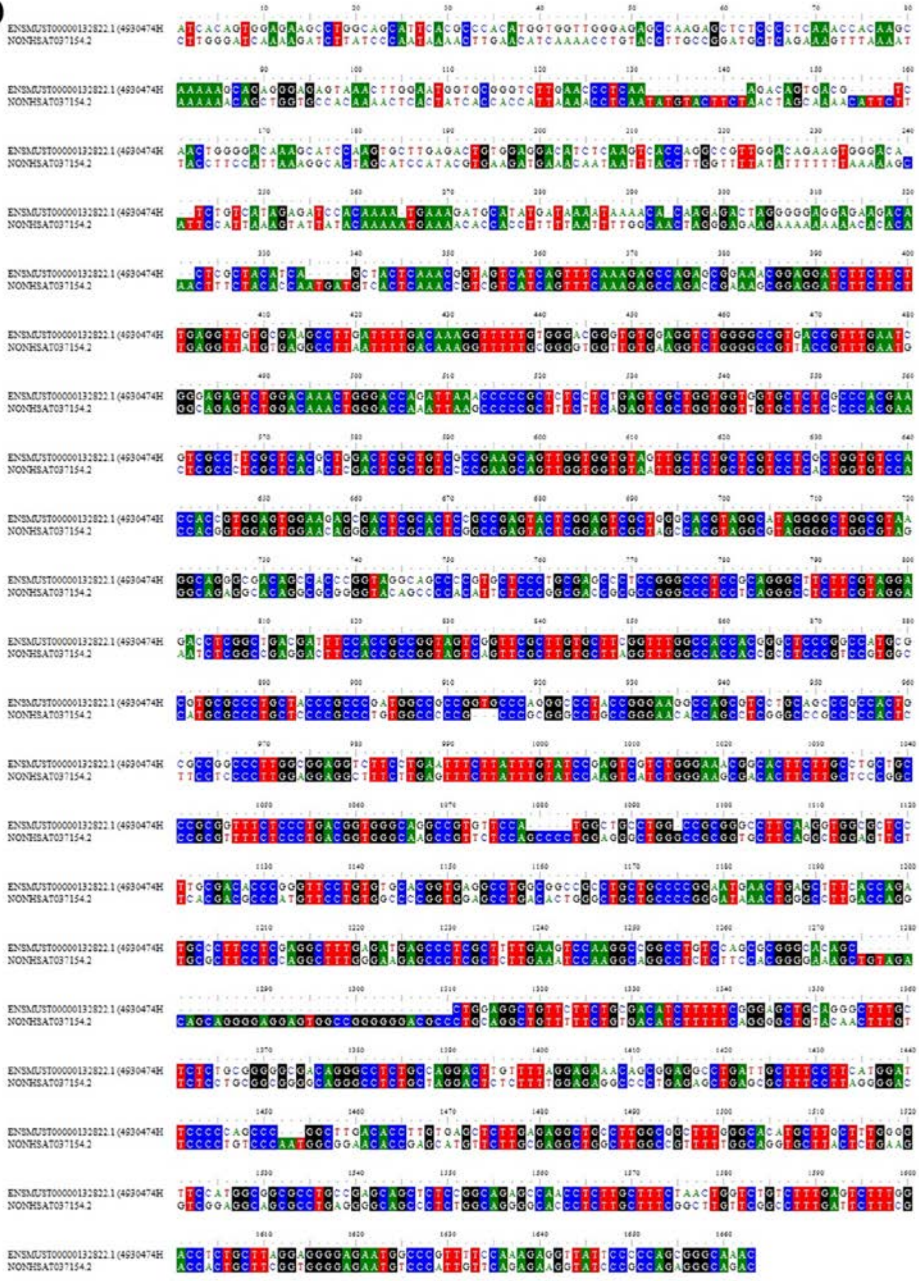

Supplementary Fig. 3. Analyses of the Gene Importance Calculator (GIC, www.cuilab.cn) and sequence alignment. (**a**) GIC analysis of ENSMUST00000132822 (4930474H06Rik) and ENSMUST00000139621 (1700028N14Rik). (**b**) Alignment of ENSMUST00000132822 (4930474H06Rik, mouse) and NONHSAT037154 (human) sequences.

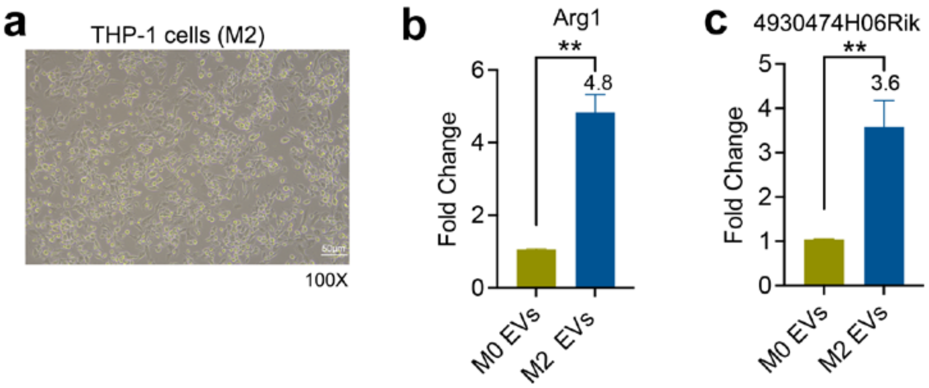

Supplementary Fig. 4. Expression of 4930474H06Rik in EVs of THP-1 cells. (a) Macrophages were induced using THP-1 cells treated with phorbol 12-myristate 13-acetate (PMA) (500 ng/mL, 48 hours) and then stimulated with IL-4 (20 ng/mL) and IL-13 (20 ng/mL). After 72 hours of stimulation, the morphology of THP-1 cells (M2) was observed by microscope. **(b)** Analyses of the expression of the M2-associated genes in M0 and M2 EVs (THP-1 cells) by RT-qPCR. **(c)** Analyses of the expression of the 4930474H06Rik in M0 and M2 EVs (THP-1 cells) by RT-qPCR. The data are representative of two independent experiments and are presented as mean ± SEM. **P < 0.01 analysed by two-tailed Student's *t* test for single comparison.

.

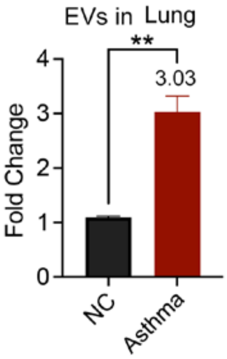

Supplementary Fig. 5. Expression of 4930474H06Rik in BMDMs and EVs in lung of asthamtic mice. Expression of 4930474H06Rik in EVs of lung tissues were detected by RT-qPCR; n = 3 mice for each group. The data are representative of two independent experiments and are presented as mean ± SEM. **P < 0.01 analysed by two-tailed Student's *t* test for single comparison.

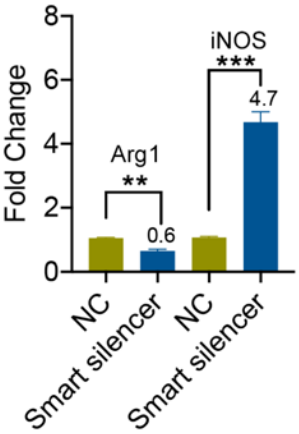

Supplementary Fig. 6. Expression of Arg1 and iNOS in M2 EVs after the down-regulation of 4930474H06Rik. Primary BMDMs were transfected with NC or 4930474H06Rik smart silencer for 48 hours, followed by stimulation with IL-4 (20 ng/mL) for an additional 48 hours. RNA was extracted from two groups of cells and subjected to RT-qPCR analysis with primers specific to Arg1 and iNOS. GAPDH was set as the endogenous control. The data are representative of two independent experiments and are presented as mean ± SEM. and analysed by two-tailed Student's *t* test for single comparison. **P <0 .01, ***P < 0.001 analysed by two-tailed Student's *t* test for single comparison.

Supplementary Fig. 7. Unedited full blots for Fig. 2c.

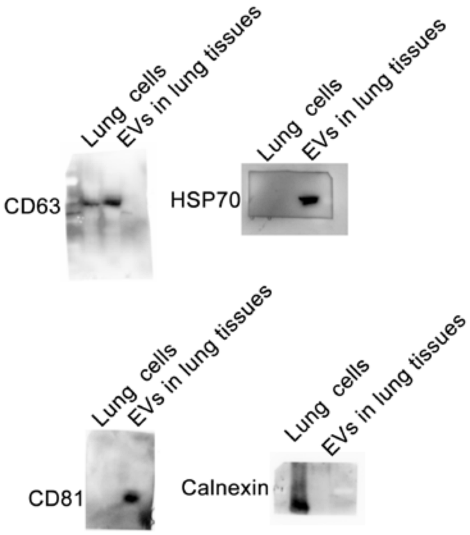

Supplementary Fig. 8. Unedited full blots for Fig. 7f.

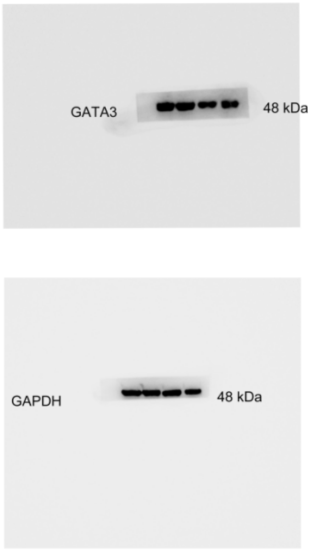

Supplementary Fig. 9. Unedited full blots for Supplementary Fig. 2e.

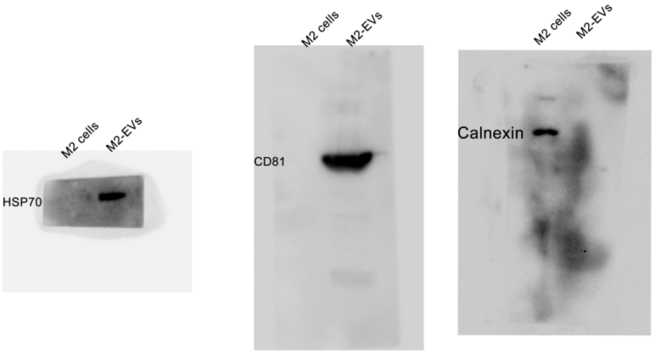

Supplement: Supplementary file 1 — Supplementary information [file 12276_2025_1465_MOESM1_ESM.pdf]
